# An optimized ensemble framework for machinery fault detection in IoT environments

**DOI:** 10.1038/s41598-026-40335-7

**Published:** 2026-02-24

**Authors:** S. V. Devi Gayadri, G. Kanagaraj, Jayant Giri, Mohammad Kanan

**Affiliations:** 1 Department of Mechatronics Engineering, Thiagarajar College of Engineering, Madurai, 625015 Tamil Nadu India; 2 Department of Mechanical Engineering, Yeshwantrao Chavan College of Engineering, Nagpur, India; 3 Division of Research and Development, Lovely Professional University, Phagwara, India; 4 Centre for Research Impact & Outcome, Chitkara University Institute of Engineering and Technology, Chitkara University, Rajpura, 140401 Punjab India; 5 Department of Industrial Engineering, College of Engineering, University of Business and Technology, Jeddah, 21448 Saudi Arabia; 6 Department of Mechanical Engineering, College of Engineering, Zarqa University, Zarqa, Jordan

**Keywords:** Fault detection, Machine learning models, Industrial machinery, Sensors, Reliability, Enhanced feature extraction and optimization, Energy science and technology, Engineering, Mathematics and computing

## Abstract

Fault detection in IoT-enabled machinery involves identifying defects in the operation of industrial equipment to foil breakdowns that ensure reliability. It typically relies on analysing data from IoT sensors that monitor key parameters such as temperature, vibration, pressure, and speed in machinery. Fault detection systems in industrial settings often face challenges due to the inconsistent distribution of critical sensor data, affecting monitoring systems reliability. Thus, this research aims to design an Optimized Robust PCA-based Ensemble framework for Machine Learning model to find the abnormalities in the IoT-enabled machinery and ensure robust performance by analysing the distribution patterns of critical sensors. This frameworkA is used to extract the essential samples based on voltage, speed, temperature and vibrations. The ensemble learning model includes K-Nearest Neighbour (KNN) and Adaboost, which are optimized by Bayesian optimization. The importance of optimization technique used to fine-tune the parameters of ensemble learning model. The improved ensembled learning model detects the fault in the IoT enabled machinery can be ensured by the performance metrics such as accuracy, precision, and detection rate and false negative rate. The effectiveness of the proposed model is evaluated by varying operational conditions and viability is analysed using an extensive dataset is IoT enabled machinery operations. Thus, the experimentation of the proposed model significantly improves fault detection reliability, ensuring operational efficiency and reducing downtime in industrial infrastructures.

## Introduction

Machinery is the backbone of industrial control systems, driving efficiency, precision, and reliability across various operations. In the era of IoT-based environments, industrial machinery has evolved to integrate smart sensor networks, enabling real-time monitoring and enhanced decision-making. These advancements have significantly improved the ability of machines to perform complex tasks with greater accuracy and consistency. However, the optimal performance of IoT-enabled systems depends on the proper functioning of critical factors such as operating temperatures, sensor accuracy, and vibration control^1,2^. IoT sensors play a pivotal role in maintaining these parameters by continuously monitoring operating conditions and transmitting data for analysis. For instance, maintaining optimal temperatures is crucial to prevent overheating, which can lead to severe damage or reduced equipment lifespan. Real-time data from temperature sensors allows for proactive intervention to avoid potential failures. Similarly, IoT-based vibration sensors can detect anomalies caused by misaligned or deteriorating components, ensuring timely maintenance and preserving precision in operations. Faults in these critical areas can lead to cascading effects such as unplanned downtime, increased maintenance costs, and reduced productivity. In IoT-enabled industrial systems, addressing these faults with advanced fault detection and predictive maintenance technologies is essential for ensuring operational efficiency, minimizing disruptions, and maintaining overall profitability^4–6^.

Despite significant advancements in fault detection, existing studies in industrial systems have revealed several limitations. Many studies have focused on specific fault types, such as bearing failures or vibration analysis, often neglecting a comprehensive approach that integrates multiple fault parameters. Additionally, traditional methods, like time-domain and frequency-domain analysis, struggle with accurately diagnosing machinery faults in complex and dynamic industrial infrastructure^3,4,7^. Another limitation is the reliance on handcrafted feature extraction techniques, which require extensive domain knowledge and may not capture subtle patterns indicative of impending failures. Furthermore, the impact of external factors like varying operational conditions, sensor noise, and environmental influences is often underexplored, leading to potential inaccuracies in fault detection^8,9^. As a result, there is a growing need for an advanced model tailored for fault analysis. In recent years, intelligent detection systems have become crucial across various fields, with Machine Learning models demonstrating exceptional performance in this domain.

Machine Learning models are effective in the aspect of classification and detection. Enormous research showcased the usage of those models in identifying faults in industrial machinery. Hence, this research article aims to develop a machine learning-based fault detection system that ensures effective detection for faults in machinery in industrial infrastructure^10–12^. Models such as Support Vector Machines (SVM), Decision Trees (DT), Random Forests (RF), and k-nearest Neighbours (KNN) have been extensively explored for fault classification and anomaly detection. These models are known for their simplicity, interpretability, and robustness in detecting faults across various operational conditions. Hence, various machine learning models have been proposed for fault detection in machinery components^13,14^. However, each models pose difficulties in multi class classification. Therefore, to overcome the issues, an optimized robust PCA-based ensemble learning model is proposed. The major contribution of the model is highlighted below,

- An optimized robust PCA technique is utilized to extract high-quality features from noisy and high-dimensional industrial machinery data.
- Stage-wise feature selectors improve multiclass detection by identifying the most pertinent features at each phase of the detection process, thereby improving the accuracy and efficiency of sensor devices.
- Ensemble model (KNN + Adaboost) provides adaptability and resilient predictive capabilities for diagnosing faults in IoT-enabled settings.
- The integration of KNN for localized decision-making with the adaptive features of AdaBoost provides an effective strategy for fault detection.
- Bayesian optimisation effectively navigates the hyperparameter space, thereby minimising the number of iterations and computational resources required.

The structure of the article is as follows: section “Related works” deliberates the recent research in advancements towards fault detection. Section “Proposed methodology” provides a detailed explanation of the proposed model. Section “Experimental details” delineates the experiment details. Section “Results and discussion” discusses the experimental results and their implications, while section “Conclusion” concludes the paper and outlines potential directions for future work.

## Related works

This section briefly discusses the recent studies on fault detection in industrial machinery that demonstrate significant advancements through Machine Learning (ML) techniques, focusing on enhancing operational reliability and efficiency. Various approaches leverage ensemble models, deep learning, and IoT-based systems to analyse sensor data and detect faults in the machineries. Owing to this, some pertinent works are discussed below, along with their limitations and scope for the proposed model.

Dhiraj Neupane et al. integrate machine learning approaches^13^ for early equipment fault detection. It emphasizes how modern sensors and big data can improve diagnostic capabilities. However, its inability to manage sensor noise and adjust to various operating circumstances limits its dependability in dynamic situations. Moshrefi et al.^14^ use ensemble machine learning models (gradient boosting and stacking) with ultrasonic signal processing for flaw detection. Though accuracy is increased using ensemble models, the research does not include real-world deployment scenarios, and computational efficiency is unaddressed. Basangar et al.^15^ tackle defects in rotating industrial gear with a deep learning focus. Despite increasing accuracy, low-resource resources are considered industrial contexts and are limited by their high computing cost and need for huge datasets. Wei Cui et al.^16^ blend methods such as Deep Belief Networks, Autoencoders, Convolutional Neural Networks, Recurrent Neural Networks, Transfer learning, and Generative Adversarial Networks. Though the model’s scalability is good, the proposed model failed to examine the effects of changing operating circumstances and inconsistent sensors. Laaradj et al.^17^ use ML to classify faults in spinning machinery using vibration signals. Although the approach shows better results, its limited feature extraction capabilities make it difficult to uncover small flaws. The significance of feature engineering and selection in enhancing ML model performance for fault detection is examined in this work. However, manual feature extraction makes model deployment more complex and calls for domain expertise.

Sohaib et al.^18^ apply advanced machine learning and image processing techniques for gearbox fault detection, achieving high accuracy (96%) with innovative methods like edge detection and maximized pooling. The study is based on vibration signal images, offering insights into using visual data for diagnostics. However, it is limited to predefined datasets and specific machinery types, reducing generalizability to other industrial applications. Qiu et al.^19^ reviews the use of deep learning models, including emerging architectures like transformers and graph neural networks, for fault diagnosis and prognosis in industrial systems. While it highlights the advancements in feature representation and fault detection, challenges like data imbalance, multimodal data integration, and edge device implementation remain unaddressed. Moosavi et al.^20^ integrate explainable artificial intelligence (XAI) approaches with traditional machine learning for fault detection, improving interpretability and trust in model predictions. Despite the promising results, these models’ computational complexity and dependence on huge datasets are identified as challenges. Perumal et al.^21^ explored the application of supervised machine learning models for detecting faults in industrial machinery. It highlights techniques for processing IoT sensor data, optimizing detection performance, and addressing challenges related to data imbalance and noise interference​. Priya et al. Analysed fault detection in wireless sensor networks leveraging machine learning algorithms to enhance fault tolerance and system reliability^22^. Leite et al. introduced an AutoML-based real-time fault detection and diagnosis system optimized for IoT environments. The^23^ system leverages discrete-event and continuous-variable features for comprehensive fault detection. Ellah et al. Explained machine learning methods for fault detection in wireless sensor networks, which are fundamental to IoT environments. It focused on techniques that prove the reliability and accuracy of fault detection mechanisms, addressing constraints like limited computational resources and varying environmental conditions^24^. Inyang et al.^25^ proposed a deep blended ensemble learning model that integrated the optimized signal transformers. A compound dataset is used for training the model to diagnose the faults in rotating machines like gearboxes, bearings and shafts. Miao et al.^26^ focused on a feature-driven ensemble learning method that integrated time and frequency domain information for fault detection in industrial processes. Bayesian inference combines results from feature extraction layers, achieving superior performance on the Tennessee Eastman Process dataset. Jose et al.^27^ proposed ensemble learning approach for bearing fault detection. Various algorithms are considered for observation among KNN, XGBoost and SVM models are considered and ensemble for fault detection. Siddique et al.^28^ proposed a Burst Aware Adaptive Frame Segmentation based Weighted Stacked ensemble model for Acoustic emission and the model achieved 99.21% of accuracy. However, the authors failed to mention the overfitting issues, computational complexity issues and also the dataset reliability is not verified. Siddique et al.^29^ proposed fault detection model with transformers based semi-supervised model and achieved and accuracy rate of 99.68%. The model was tested using the benchmark dataset and the real time valuation is not discussed which lead to the limitations of data reliability. Zaman et al.^30^ proposed a hybrid framework for fault detection mechanism using logarithmic continuous wavelet scalograms, Canny edge operator, and dual branch encoder. Despite this framework, it has limitations such as dataset reliability, limited generalizability, overfitting issues and complexity in tuning the parameters of the proposed models. Zahoor et al.^31^ proposed a framework for fault diagnosis of centrifugal pumps using various methods however failed to mention the uncertainty of other fault types and pumps.

The reviewed works highlighted the significant advancements in fault detection using machine learning techniques for industrial machinery. However, several limitations persist across these studies (i) some models failed to address sensor noise and dynamic operating conditions, reducing their approach’s adaptability and reliability. (ii) Enhanced accuracy with ensemble models, overlook computational efficiency and real-world deployment feasibility. Overall, these limitations underline the need for models that are computationally efficient, generalizable, and robust to diverse conditions.

## Proposed methodology

**Fig. 1:**
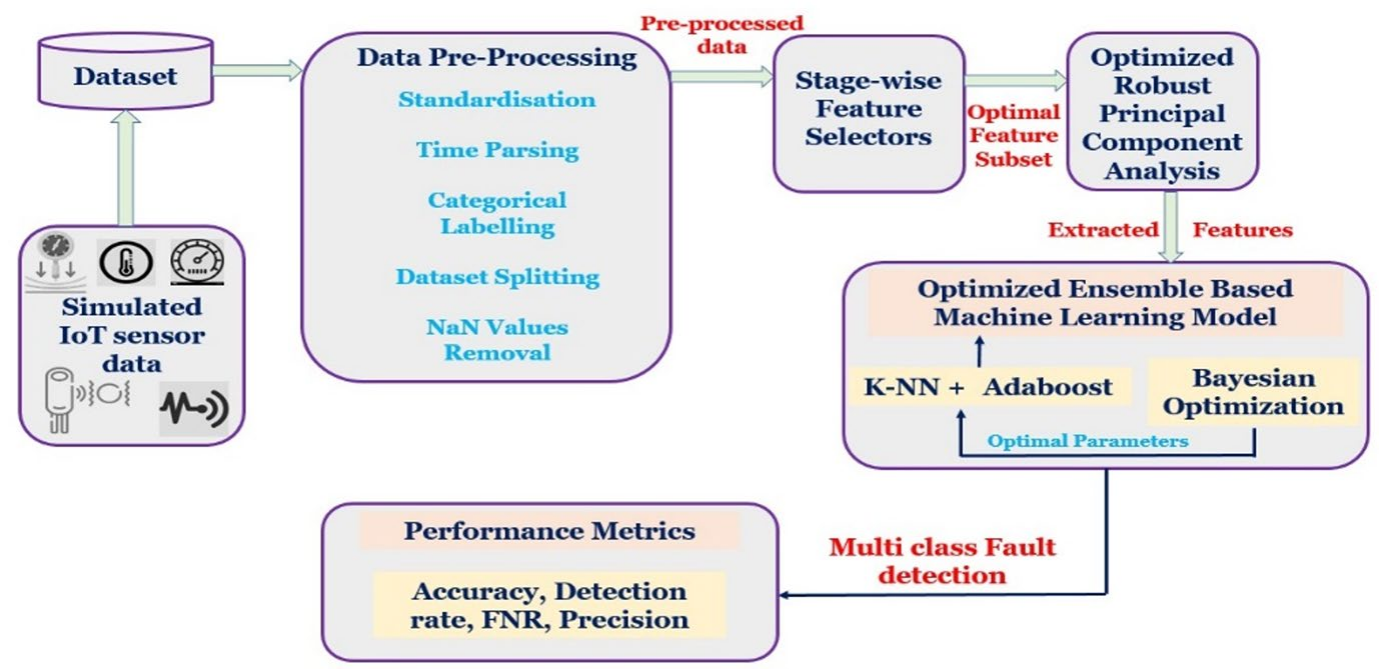
Proposed framework of optimized RPCA and ensemble learning model.

The proposed framework of Optimized RPC and Ensemble learning model shown in Fig. 1 is used to detect faults in industrial machinery and prevent further defects. Though various research scopes focus on ensemble-based solutions, most of the works failed to address the problem of computational efficiency, pre-processing model, data dependencies, and scalability. To address all these issues, an **optimized Robust Principal Component Analysis based Ensemble Machine Learning model (R-PCA based EML)** is proposed and validated using real-time data collected from the simulated environment for a period. The working of the proposed method is categorized into three subsections: (i) Data Pre-processing, (ii) Feature selection & Feature extraction, and (iii) Optimized Ensemble Machine Learning based fault detection model and discussed in the following subsections.

### Data collection

The dataset is collected using a real-time IoT monitoring simulated environment where wireless sensors are strategically deployed to continuously monitor critical machine parameters, including Temperature, Pressure, Speed, Power Supply Voltage, and Vibration. These parameters provide real-time insights into the machine’s operational state, serving as a foundation for fault detection and health analysis. Each wireless sensor captures relevant data and transmits it wirelessly to the Wireless Sensor Network (WSN) module, which acts as a central hub. The WSN module relays the aggregated sensor data to a MyRIO-embedded device, which interfaces with a personal computer for further processing and analysis. Wireless communication ensures seamless monitoring, even in hazardous or remote environments, making the system highly adaptable to various industrial applications. The data captured by the sensors is timestamped and recorded using NI LabVIEW programming software. This platform provides a robust, flexible environment for real-time data acquisition, analysis, and visualization. The timestamped data is stored alongside the current date and time, facilitating historical analysis and trend identification. This is critical for predicting potential machine failures. By maintaining a comprehensive log of machine parameters, the system enables engineers to: (i) Analyse the machine’s long-term performance and (ii) Detect gradual trends that may indicate future malfunctions. This systematic approach to data collection ensures a reliable and scalable framework for proactive machine monitoring and fault detection. The major motivation for the simulation of this dataset relies on systematically controlling the fault types, severity levels in the machinery systems, and operation managing sensors. This dataset ensures the consistency and generate the sufficient fault instances replicating the architect and statistical alignment of real time industrial environment.

**Fig. 2:**
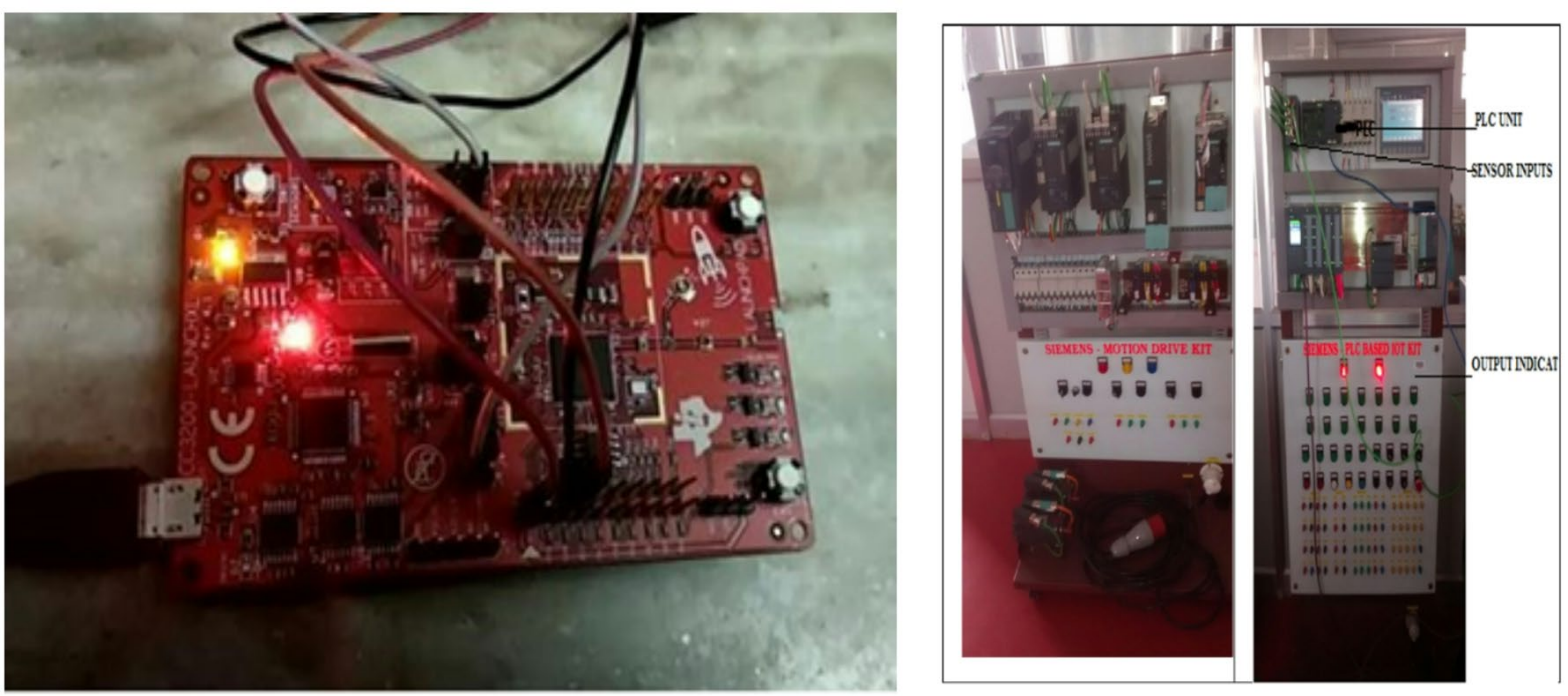
(a) CC3200 WiFi Launch, (b) motion drive Kit and PLC based IOT Kit.

The sensors are interfaced with the WiFi Module Kit through a breadboard and connecting wires. The WiFi Module Kit [Fig. 2 (a) and (b)] is initially connected to a PC system for program development, including writing, compiling, and uploading. Once the program is successfully uploaded to the module, it is disconnected from the PC and prepared for data acquisition.

### Data pre-processing

Data pre-processing is imperative for training the model to handle sensor-based data effectively. These data contain null values, outliers, and inconsistencies, which degrade the learning model’s performance. Hence, to ensure the reliable analysis of data, pre-processing is crucial. It includes data cleaning, data integration, data transformation, and so on. In our proposed model, we have included various data pre-processing techniques, which are described as follows,

- **Standardisation**

The data has been rescaled with the mean value of 0 and the standard deviation of 1^30^. This is also called z-score scaling. The process is carried out using the formula given in formula (1).

1 $$sf'=\left( {of - mean\left( {of} \right)} \right)/\left( {sd\left( {of} \right)} \right)$$

Where $sf'$ denotes standardised feature, $of$ original feature and $sd\left( {of} \right)$ refers the standard deviation of original feature.

(b) **Time parsing**

As most IoT sensor data consists of “date” and “time” as columns, this is converted to a single timestamp value or date-time format for consistency and easier indexing.

(c) **Categorical labelling**

Considered simulated IoT sensor data has features “vibration”, “speed”, “voltage,” and “temperature.” And their relative values. There are no specific labels for fault analysis. As per the domain and expertise knowledge, some criteria are framed for fault analysis (A detailed explanation of these criteria is given in the data description section). The labels are as follows: **“Very Low (VL)”**,** “Low (L)”**,** “Medium (M)”**,** “High” and “Very High”.**

(d) **Dataset splitting**

Data splitting is an important process in the machine learning process as it helps in ensuring the model to avoid overfitting and under fitting^28^. Hence, the data is splitted as **training (80%) and testing data (20%).** The model is trained using the training data and the testing data is unseen data. The model is robust when it performs well with testing data.

(e) **NaN values removal**

NaN values, or “Not a Number,” play a crucial role in data analysis as they indicate missing or incomplete information in a dataset^29^. Effectively managing these values is essential to maintain the accuracy and reliability of analytical results. We used ***“isnan()”*** function from the python library to manage the NaN values in the considered dataset.

### Feature selection

Feature selection is a crucial step in pre-processing, particularly for datasets containing multiple correlated attributes^31,32^. In this article, we propose a **Stage-Wise Feature Selection** methodology to identify optimal subsets of features that contribute significantly to fault detection in machinery. By adopting this method, we aim to enhance model performance and interpretability while reducing computational overhead. The feature selection process is structured into distinct stages, each evaluating a specific subset of the dataset. The subsets are constructed based on the combination of the timestamp feature, which provides temporal context, with other key attributes that represent critical operational parameters of the machinery. These stages are described in Table 1.

**Table 1:** Four different stages of IoT enabled machinery dataset.

Stages			
Stage 1 (Voltage Vs Timestamp)	Stage 2 (Speed Vs Timestamp)	Stage 3 (Temperature Vs Timestamp)	Stage 4 (Vibration Vs Timestamp)
This subset focuses on the relationship between temporal changes and voltage variations. Voltage serves as a critical indicator of electrical stability and faults, making it vital for detecting anomalies in electrical systems	This stage evaluates the interplay between time and speed, representing dynamic operational states of the machinery. Variations in speed over time can signify mechanical imbalances or performance degradation	The third subset examines the correlation between time and temperature. Temperature variations often reflect thermal stress or overheating, which are precursors to machinery failure	The final stage considers temporal data alongside vibration measurements. Vibration is a key diagnostic parameter for identifying mechanical wear and tear or misalignments

By segmenting the dataset into these targeted feature subsets, the stage-wise selection method allows for a comprehensive analysis of how each parameter contributes to fault detection. This iterative approach ensures that the most relevant features for fault diagnosis are retained, while redundant or less significant features are excluded, thereby improving the model’s diagnostic capabilities.

### Feature extraction—optimized robust principal component analysis

After feature selection, feature extraction becomes a pivotal step in preparing the dataset for fault detection tasks. Feature extraction reduces the dimensionality of the dataset while preserving the most informative components, ensuring the model’s efficiency and robustness. Hence, an optimization-based Robust Principal Component Analysis (R-PCA) method^33–35,37^ is proposed to extract critical features from the selected subsets of the dataset.

RPCA decomposes the data matrix *x* into two components: one as low rank matrix $LM$ which represents the underlying patterns of the data. Whereas the sparse matrix is represented as $SM$ which captures the anomalies. This decomposition process is performed by solving the optimization problem in formula (2)

2 $$\mathop {\min }\limits_{{LM,SM}} \left| {\left| {LM} \right|} \right|_{*} + ~\lambda \left| {\left| {SM} \right|} \right|_{\prime } {\mathrm{~subject~to~}}x = LM + SM$$

Where ${\left| {\left| {LM} \right|} \right|_*}$ denotes the nuclear form of$LM$. $\left| {\left| {SM} \right|} \right|_{\prime }$ represents the ${l_1}$ normalization of $SM$. The parameter λ controls the trade-off between the low-rank approximation and the sparsity of the noise component. The following subsection will explain how the R-PCA works with the considered machinery dataset.

### Optimized ensemble machine learning based fault detection model

Industrial systems rely heavily on robust fault detection mechanisms to ensure machinery operates efficiently without unexpected breakdowns. Fault detection involves identifying abnormalities that lead to system failures and to ensure timely intervention. With advancements in machine learning, ensemble models have become a preferred choice for tackling complex fault detection problems. Hence, we proposed an optimized ensemble machine learning model combining K-Nearest Neighbours (KNN) and AdaBoost classifiers. The model’s performance is further enhanced through Bayesian Optimization, which fine-tunes hyper parameters of ensemble models, leading to improved multiclass fault detection.

KNN is a simple, non-parametric machine learning algorithm widely used for classification problems, including fault detection. The primary concept behind KNN is that similar data points exist close to each other in the feature space^36^. The algorithm assigns the label of the majority class among the nearest neighbours to a data point under consideration. AdaBoost (Adaptive Boosting) is an ensemble learning algorithm that combines multiple weak classifiers to create a strong classifier. In the context of fault detection, AdaBoost iteratively adjusts weights for data points, emphasizing those that were misclassified in previous iterations^38–40^.

The proposed model combines KNN and AdaBoost predictions through a weighted voting mechanism. KNN Captures localized patterns and provides probabilistic outputs for each class based on the nearest neighbours. While, AdaBoost Contribution focuses on hard-to-classify instances and assigns higher confidence to classes that are challenging for detection process. Subsequently, weighted voting aggregates these predictions, ensuring that each classifier’s strengths are utilized. The final decision is based on the class with the highest aggregated score. Meanwhile the ensemble model’s parameters are tuned using the Bayesian optimization process.


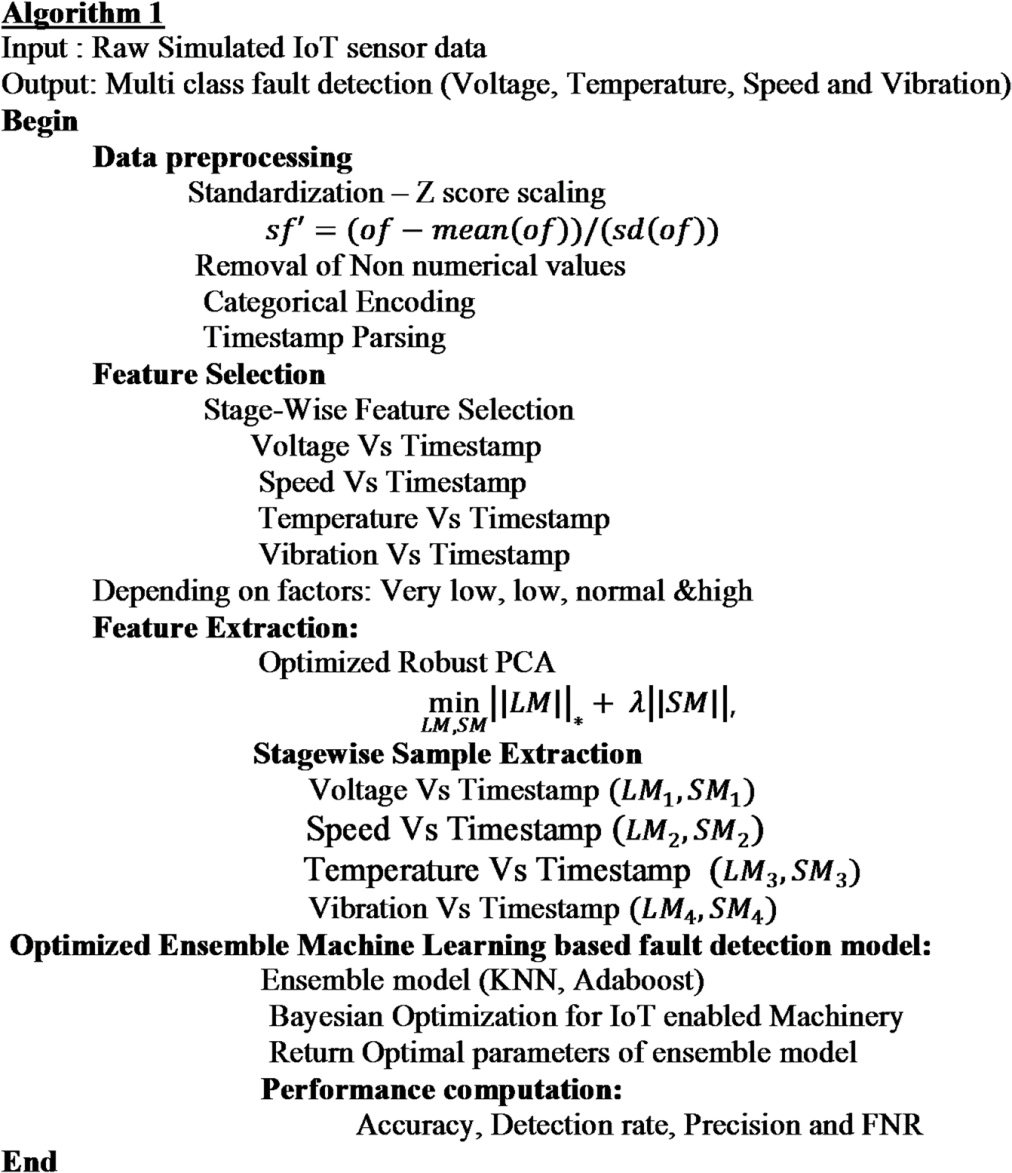


### Optimization process—Bayesian optimization

**Fig. 3:**
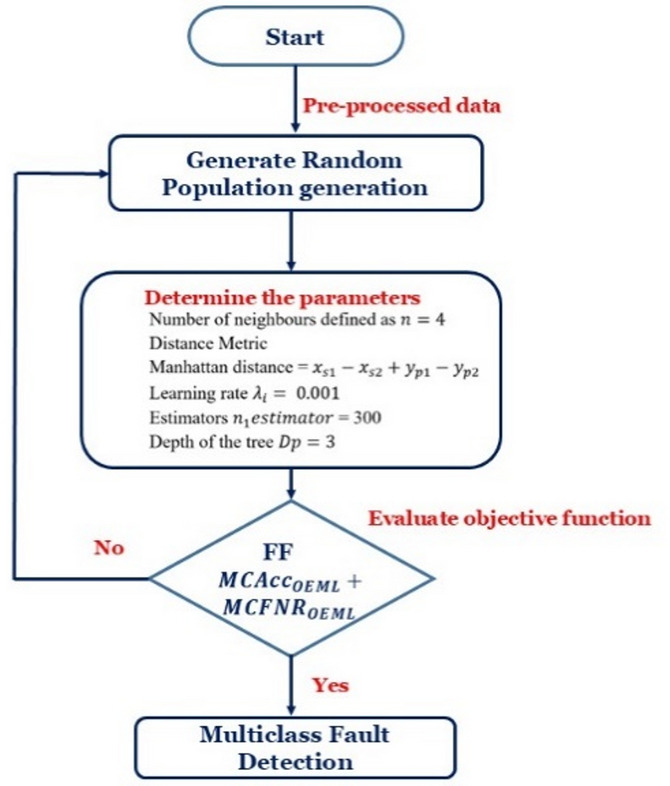
Working of Bayesian optimization-based ensemble learning model.

Hyper parameter tuning plays a critical role in improving the performance of machine learning models. Manual or grid-based tuning is inefficient and time-consuming whilst the Bayesian Optimization offers a systematic and efficient way to identify optimal hyper parameters by modelling the objective function and iteratively refining the search space. The working procedure of the Bayesian Optimization is shown in flowchart of Fig. 3.

Bayesian Optimization involves constructing a probabilistic model, typically a Gaussian Process (GP), to approximate the objective function. Based on this model, the algorithm identifies hyper parameter configurations that yields optimal results. The parameters of KNN and Adaboost considered for optimization process are tabulated in Table 2 (i) Number of Neighbours, (ii) Distance Metric – Manhattan, (iii) Learning rate (), (iv) Estimators and (v) Depth of the tree. The iteration process ends once the fitness function is reached.

**Table 2:** Bayesian parameters & its values.

Parameters	Values
Number of neighbours	$n=4$
Distance Metric (Manhattan distance)	${x_{s1}} - {x_{s2}}+{y_{p1}} - {y_{p2}}$
Learning rate	${\lambda _i}=~0.001$
Estimators	${n_1}estimator=300$
Depth of the tree	$Dp=3$

The following formula (3) specifies the objective function considered for multiclass fault detection

3 $$Fitness~Function=MCAc{c_{OEML}}+~MCFN{R_{OEML}}~$$

Where the $MCAc{c_{OEML}}$ denotes the aggregated accuracy of multi class fault detection, $MCFN{R_{OEML}}$ mentions the aggregated False Negative Rate. These two metrics plays the significant role in fault detection process and upholds the learning model’s performance; it represents the accurate detection and the rate of negative samples which are wrongly predicted.

## Experimental details

This section demonstrates the analysis carried for fault detection in IoT enabled machinery. The model is evaluated using simulated IoT enabled machinery dataset. The following subsection provides the detailed data description. The proposed model encompasses data collection followed by stage wise feature selectors which is processed by imposing the timestamp feature which is common to correlate with all the features and the model produce feature subsets based on the timestamp. Then, Optimization concept is implemented for feature extraction and detection method. Optimal Features are extracted from the R-PCA method using the tuning concept. λ Parameter is tuned to manage the low rank and sparse PCA components. Also, the ensemble models are optimised using the Bayesian optimization method by tuning the specific parameters of learning model which enhances the performance and produce the accurate results. The experiments analyse few performance metrics while examining computational efficiency, interpretability, and feature importance. The results provide insights into model selection and optimization strategies, emphasizing the trade-offs between complexity, accuracy, and application requirements.

### Experimental setup

The proposed optimized Ensemble learning model was implemented in python 3.5 with the TensorFlow framework and keras library was used for evaluation process. The experimentation was conducted on labelled Industry based IoT enabled machinery and performed on intel core i7 processor with 16 GB RAM running Windows 11 OS.

### Dataset description

The dataset comprises sensor data recorded using NI LabVIEW programming software, which provides a flexible and robust platform for real-time data acquisition, analysis, and visualization. Each entry in the dataset is timestamped with the current date and time, allowing for historical analysis and the identification of trends critical for predicting potential machine failures. The dataset includes 3608 samples with seven columns: Date, Time, Voltage, Speed, Vibration, Temperature, and Pressure. The voltage readings range from 220 to 250 volts, capturing fluctuations in electrical supply. Speed measurements span from 1000 to 3000 m/s, reflecting the operational dynamics of the machine. Vibration data, ranging between 300 and 600 m/s², provides insights into mechanical stability and potential anomalies. Temperature values range from 15 to 55 degrees Celsius, enabling monitoring of thermal conditions that may indicate overheating or suboptimal operating environments. Pressure values fall between 80 and 160 Pascals, highlighting variations that may impact system performance. This dataset presents a comprehensive log of machine parameters, facilitating long-term performance analysis and enabling engineers to identify gradual trends or faults in machineries that may lead to future malfunctions.

### Data pre-processing & feature engineering

**Fig. 4:**
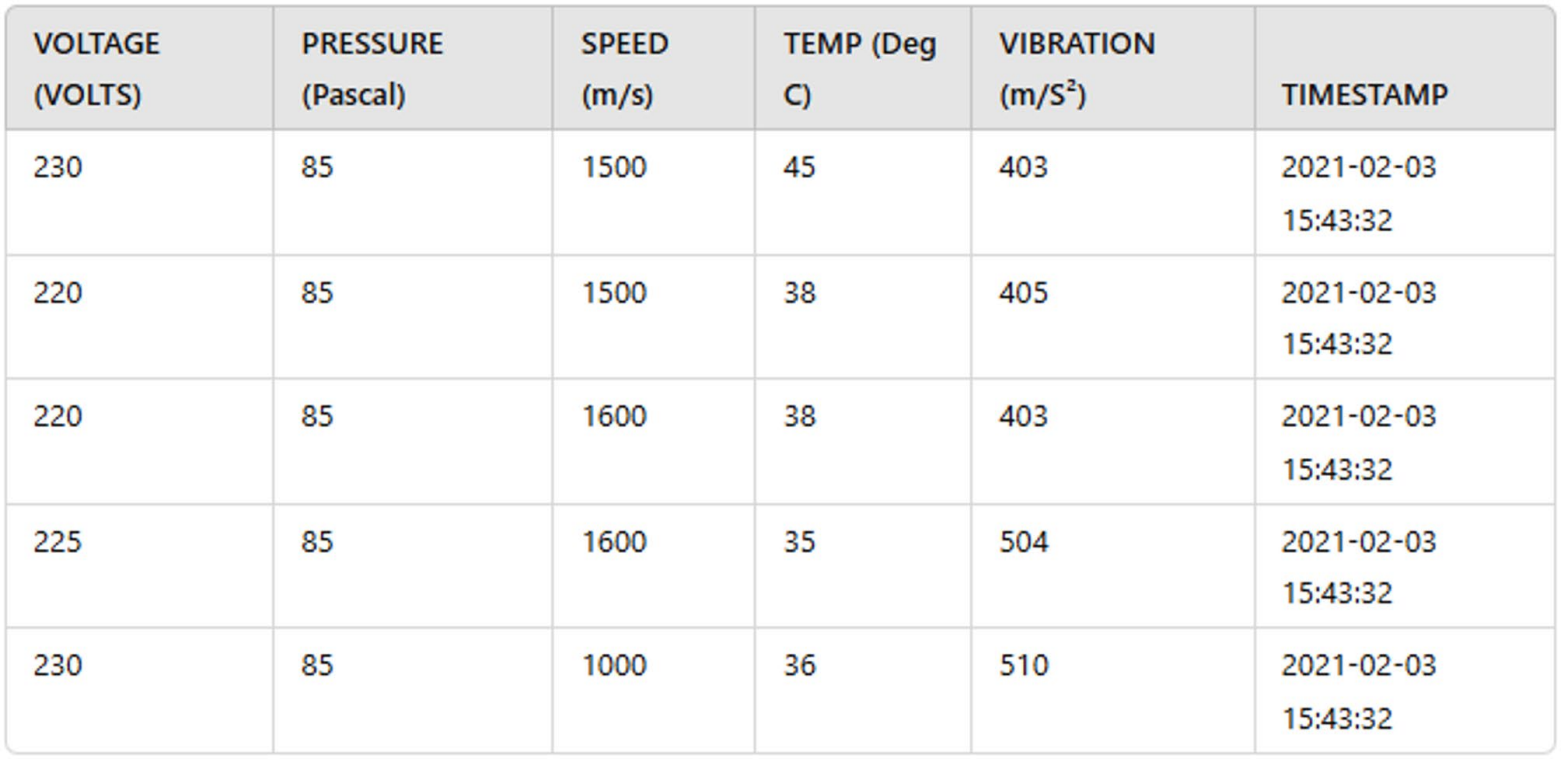
Sample data after time parsing.

The collected data is pre-processed before training the model for fault detection in IoT enabled machinery. The process included to pre-process the data are data cleaning, integration and transformation. The standardization of z-score scaling is applied to scale the values of the attributes of voltage, pressure, speed, temperature and vibration. For conversion of date and time of dataset as single column, the method of time parsing is deployed. Figure 4 represents the time parsing in the IoT enabled machinery data. Due to the absences of specifications especially in case of analysing the fault in the IoT enabled machinery, the categorical labelling is used to label the categories are very low, low, medium, high and very high. As per data cleaning, the dataset are cleaned by removing all unwanted and non -numerical values. Once, the data are pre-processed, the feature engineering techniques are employed to the stage wise feature selector using feature selection technique and extract the essential samples through feature extraction.

Feature selection for multi-correlated features, the stage-wise feature selection method is presented by combination of timestamp with all the attributes of dataset are voltage, speed, temperature and vibration. The obtained stage wise feature shows the subset of different stages that defines the sensitive operational mechanisms of IoT sensors.

**Stage 1: Voltage Vs Timestamp**

The voltage fluctuations are noted from 220 V to 250 V where 220 V specifies the very low, 220 to 230 turns low volt, 230 to 240 turns normal ,240 to 250 V is high and when the voltage goes greater than or equal to 250 volts, it reaches high Volt. Thus, samples which covers except these ranges are selected based on timestamp. The well working state of the machinery are coming under normal and low where normal volt is comfortable compared to low.

**Stage 2: Speed Vs Timestamp**

The speed (m/sec) of a IoT sensors are marked from 1000 to 2000 m per second. It also classified in four categories from very low to high. The speed which goes less than 1000 will makes the IoT sensor to malfunction. Similarly, the speed less than 1500 are marked as low speed that slowdown the entire process of the IoT sensors. The normal speed for the machinery with IoT sensors from 1500 to 2000 that practices the machinery with balanced speed and the speed of machinery when works greater than 2000 that shows the IoT sensor-based machinery are working out of control. So, the machinery should be worked in two cases are normal and low, where normal is possible however, low speed can be ignored.

**Stage 3: Temperature Vs Timestamp**

The temperature for the working state of IoT enabled machinery are defined in four different cases.

Case 1: very low- A temperature lies less than 15 degrees Celsius.

Case 2: low-A temperature lies between 15 and 25 degrees Celsius.

Case 3: Normal – A temperature lies from 25 to 45 degrees Celsius.

Case 4: High-A temperature from 45 to 55 & greater than 55 degrees Celsius.

When a temperature reaches very low or high state leads to thermal stress or overheating to the machinery that cause failure in machinery.

**Stage 4: Vibrations Vs Timestamp**

**Fig. 5:**
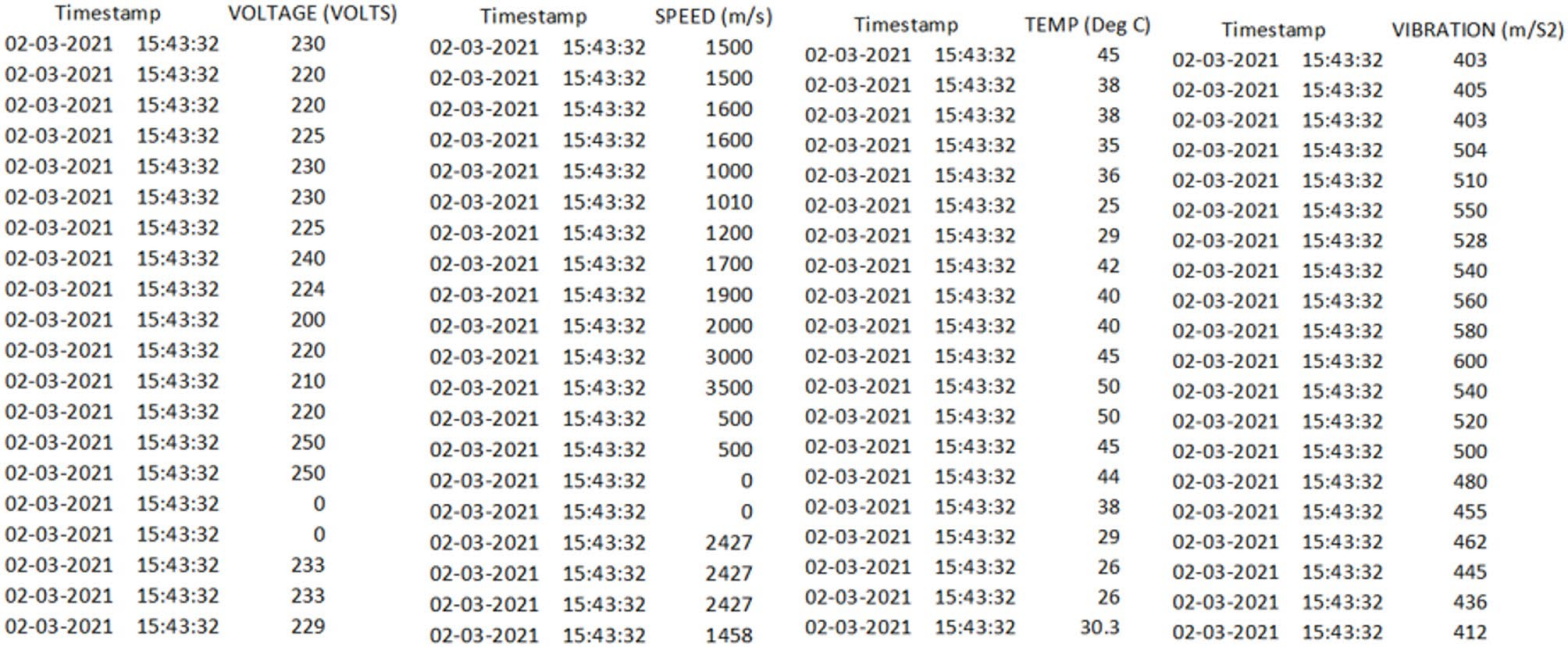
Sample stage-wise feature selectors.

It concentrates on monitoring oscillatory motion using sensors to predict the deviations from normal behaviour of the machinery. Normal state of vibrations lies from 400 to 500 that notify the steady state of IoT enabled sensors. However, the rest of the three cases are very low has less than 300, low lies between 300 and 400 and high 500 to 600.Its analysis when a specific fault occurred in the machinery. Thus, stage -wise feature selection method represents the importance of the attributes with timestamp and the four different cases for each attribute, among those cases, the monitoring the three cases (very low, low and high) in mandatory because it leads to fault the machinery in various chances. Thus, all the stage-wise feature selector are shown in Fig. 5.

### Comparison on effectiveness of the proposed stage-wise feature selector model

The proposed stage-wise feature selection model is substantially compared with the baseline models, such as PCA, Mutual Information, Relief, and Recursive Feature Elimination (RFE). The comparison is performed based on the identical experimental valuation using the same ensemble classifiers and valuation metrics.

Table 3 clearly captures the quantitative comparison analysis of the proposed stage-wise feature selection model. To ensure the fairness of all the models, the evaluation is based on the same classifier model, of which the proposed model outperforms other models with the scores 0.985 accuracy, 0.984 precision, 0.988 detection rate and 0.02 FNR. Though relief exhibits better accuracy of 0.964, it is 2% lesser than the proposed model. The method of feature extraction used for fault detection in IoT-enabled machinery is robust principal component analysis (RPCA). Table 3 presents the detailed view of the parameters taken for training the learning model. To optimize the r-PCA, the learning parameter “Lamda $\:\boldsymbol{\lambda\:}$” is tuned until the model extracts the relevant feature samples. λ controls the trade-off between the low-rank representation of data and the sparse noise matrix during dimensionality reduction. The kernel function and the number of components specifically denote the model’s effectiveness.

**Table:** 

Models	Accuracy	Precision	Detection rate	FNR
PCA	0.921	0.912	0.934	0.12
Mutual information	0.957	0.941	0.956	0.15
RelieF	0.964	0.954	0.948	0.10
RFE	0.914	0.897	0.914	0.17
Proposed feature selection model	0.985	0.984	0.988	0.02

**Table 3:** RPCA components & their description.

Component	Parameter	Value	Optimization model
RPCA	$\lambda$	Optimal values are used after training	Domain Knowledge based optimization
	Number of Components	Automatically determined based on explained variance	
	Kernel	Radial Basis Function (RBF)	

Given the machinery dataset with attributes such as timestamp, voltage, speed, temperature, and vibration, R-PCA is applied to each stage-wise subset to extract meaningful features:

**Stage 1: Voltage Vs Timestamp**: The temporal voltage patterns often reveal deviations due to electrical instability. RPCA isolates the low-rank matrix $L{M_1}$representing the nominal voltage trends over time, while $S{M_1}$ captures sudden voltage spikes or drops, which may indicate electrical faults.

**Stage 2: Speed Vs Timestamp**: Variations in machinery speed over time can signal performance degradation or mechanical issues. RPCA applied to this subset extracts ${M_2}$​, which encapsulates consistent operational speed patterns, and $S{M_2}$ identifying abnormal fluctuations.

**Stage 3: Temperature Vs Timestamp**: Temperature readings are influenced by both environmental and operational conditions. The RPCA decomposition yields ${M_3}$​, representing typical temperature evolution, and $S{M_3}$​, isolating thermal anomalies like overheating.

**Stage 4: Vibration Vs Timestamp**: Vibration data is critical for diagnosing mechanical wear or alignment issues. By applying RPCA, $L{M_4}$​ captures the inherent vibration characteristics, while $S{M_4}$​ highlights unusual patterns associated with faults.

**Fig. 6:**
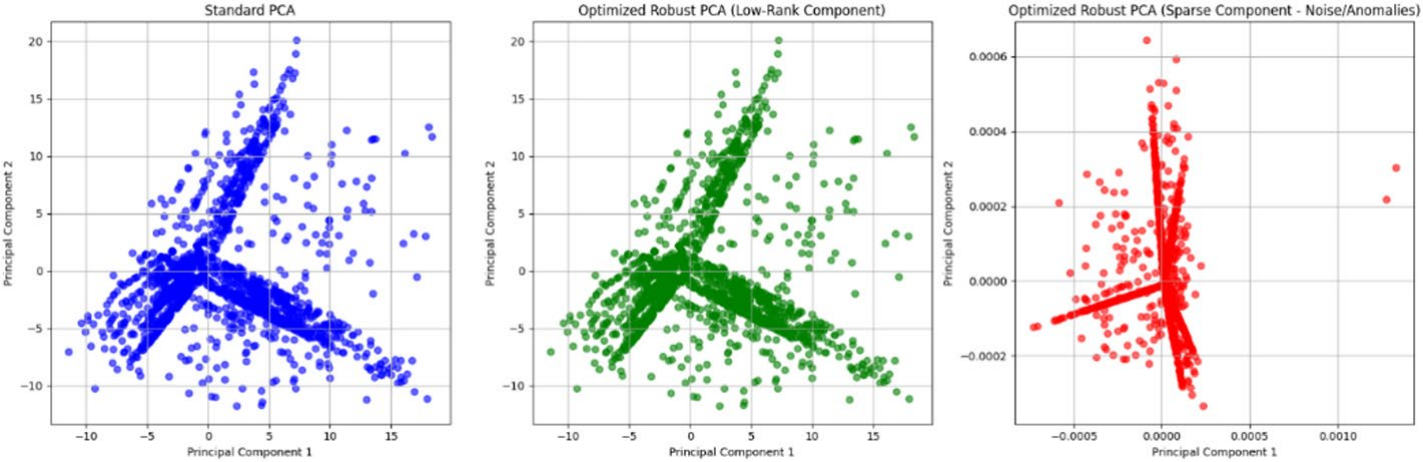
Scattered plot of Standard PCA. optimized RPCS (Low rank), Optimized RPCA (Sparse).

In Fig. 6, the blue plots from the figure denotes the Standard PCA, which projects the data onto principal components (PCs) that maximize variance. While it reveals the structure of the dataset, it is sensitive to outliers and noise, as seen by the scattered points deviating from the core data structure. In fault detection, this sensitivity may cause false alarms due to the influence of outliers or irrelevant noise. While the green plots and red plots refers to the representation of R-PCA. It separates the data into a low-rank component (core structure) and a sparse component (anomalies and noise). The plot focuses on the low-rank component, capturing the underlying normal behavior of the system while suppressing noise. The sparse component highlights noise and anomalies (data points that deviate significantly from the normal pattern). Figure 5 demonstrate how r-PCA outperforms standard PCA by providing a clearer separation of normal and anomalous data, making it highly suitable for complex industrial systems.

The extracted low-rank matrices $L{M_1},L{M_2},L{M_3},L{M_4}$​ serve as compact representations of the original data, aiding the fault detection model to focus on the most informative aspects of the machinery’s operational states. Simultaneously, the sparse matrices $S{M_1},S{M_2},S{M_3},S{M_4}$ provide actionable insights into anomalies, which are essential for pre-emptive maintenance. Optimization-based RPCA further refines this process by adaptively tuning $\lambda$ to balance the contributions of $LM~$ and $SM~$ based on the dataset’s specific characteristics. By integrating optimization-based RPCA into the feature extraction pipeline, enhances the reliability and precision of subsequent fault detection tasks, ensuring robust diagnostics and minimizing downtime in IoT environment.

To ensure the quantitative stability of the proposed RPCA method, the decomposition of the $\lambda$ is varied with the different values stating from 0.005 to 0.050. Table 5 presents the detailed quantitative analysis based on the metrics reconstruction error, sparsity ratio, Detection rate and accuracy.

Reconstruction error is defines with the normalization form of frobenius where it is expressed as Eq. (4)

4 $$\left( \mathcal{R} \right)={\left| {\left| {{\mathbb{X}} - {\mathbb{L}} - {\mathbb{S}}} \right|} \right|_{\mathbb{F}}}/({\left| {\left| {\mathbb{X}} \right|} \right|_{\mathbb{F}}}$$

${\mathbb{X}}$ represents the data matrix, ${\mathbb{L}}$ represents the low rank component, and Sparse Component ${\mathbb{S}}$. ${\mathbb{F}}$ represents the frobenius norm. The reconstruction error is evaluated in the form that low scores defines the more accurate and stable RPCA decomposition form. The sparsity ratio will measure the proportion of non-zero elements and reflects which anomalies or fault information are isolated Eq. (5)

5 $$\mathcal{S}~\left( {{\% }} \right)=\frac{{{{\left| {\left| s \right|} \right|}_O}}}{{m{\mathrm{*}}n}}{\mathrm{*}}100$$

${\left| {\left| s \right|} \right|_O}$ represents the pseudo normalization with non-zero elements and $m{\mathrm{*}}n$ represents the number of rows and columns present in the sparse matrix.

**Table:** 

$\boldsymbol{\lambda}$ Values	Reconstruction error	Sparsity ratio	Detection rate	Accuracy
0.005	0.241	9.4	0.917	0.915
0.010	0.213	8.7	0.924	0.927
0.015	0.187	8.4	0.964	0.935
0.020	0.176	7.3	0.978	0.958
0.025 (Optimized)	0.102	6.5	0.988	0.985
0.030	0.145	7.9	0.964	0.961
0.035	0.167	8.1	0.954	0.958
0.040	0.154	8.4	0.941	0.942
0.045	0.165	8.8	0.924	0.921
0.050	0.197	9.1	0.912	0.901

The results indicate that the $\boldsymbol{\lambda}{\mathrm{~}}$ parameter heavily relies on the RPCA behaviour. If the value of $\boldsymbol{\lambda}{\mathrm{~}}$parameter is less then it leads to high reconstruction errors with insufficient sparsity ration values. However, if the value is high, then it leads to less reconstruction error and fluctuations in the sparsity with low accuracy and low detection rate. Whereas, the optimized value of 0.025, achieves the better value in all the aspects with higher accuracy of 0.985, detection rate with 0.988, balanced sparsity ration as 6.5 and minimal reconstruction error with 0.102. Hence the presence of the parameter with optimised value remains the marginal, following decomposition stability and robustness.

### Fault detection in IoT enabled machinery using optimized ensemble machine learning

**Fig. 7:**
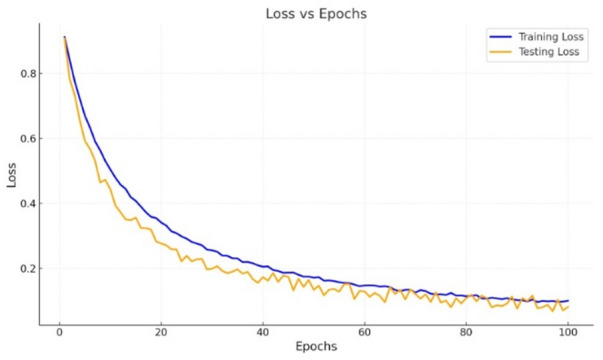
Loss Vs epoch of proposed model.

The ensembled machine learning model is the combination of KNN and Adaboost which are optimized by Bayesian optimization for fine tuning the learning rate of the ensemble learning model. While designing the ensemble learning model the parameters shown in Table 4, the higher *n* can smooth out noise but risks overgeneralizing, while a lower *n* increases sensitivity to local patterns. Setting *n*= 4 ensures that the classification balances noise reduction and sensitivity, suitable for detecting subtle fault signatures in machinery. The Manhattan distance emphasizes horizontal and vertical movements, which suits high-dimensional IoT sensor data where correlations between features might not follow Euclidean behaviour. It provides a simple yet effective metric for fault classification, especially for sensor readings like vibration, speed, and temperature. A small learning rate$~{\lambda _i}=0.0001$) ensures that weak learners do not overpower the ensemble model, allowing gradual improvements in performance. It prevents overfitting by spreading learning across multiple iterations, making the model robust to unseen data. Setting $n1=300$ ensures the ensemble model has enough complexity to capture subtle fault patterns while avoiding overfitting. A larger number of estimators improves classification accuracy for multi-class problems, such as distinguishing between vibration, speed, voltage, and temperature faults. Ωith $dp=3$ the decision trees remain simple, reducing the risk of overfitting and improving computational efficiency. Shallow trees focus on identifying strong, generalized fault indicators rather than overfitting to minor variations in the data. By tuning these parameters using Bayesian optimization, the model is well equipped and produced better results in all aspects. By computing the Mean Squared Error (MSE), the loss occurs 0.02 for testing and 0.03 for training is achieved for 100 epochs. In Fig. 7, blue denotes the training loss and yellow represents the testing loss decrease steadily as the number of epochs increases, demonstrating that the model is learning effectively. The loss reduction is significant during the initial epochs, with diminishing returns as training progresses. The testing loss is slightly lower than the training loss for most epochs. This indicates that the model has been regularized properly or benefits from a well-generalized dataset, prevents overfitting. The two curves converge toward the end, with minimal differences in loss values, which is a sign of balanced training. Both training and testing losses stabilize near 0.05 to 0.1 after 80 epochs, indicating that the model achieves low errors for both training and unseen data.

**Table 4:** Optimized ensemble learning model parameters & its descriptions.

Component	Parameter	Value	Optimization model
K-Nearest Neighbours (KNN)	Number of neighbours $\left( n \right)$	4	Bayesian optimization
	Distance metric	Manhattan distance	
	Weight function	Uniform	
Adaboost	Learning rate ${\lambda _i}$	0.0001	
	Number of estimators $n1$	300	
	Max. no. of. depth	3	
	Loss function	Modified Huber Loss	

**Fig. 8:**
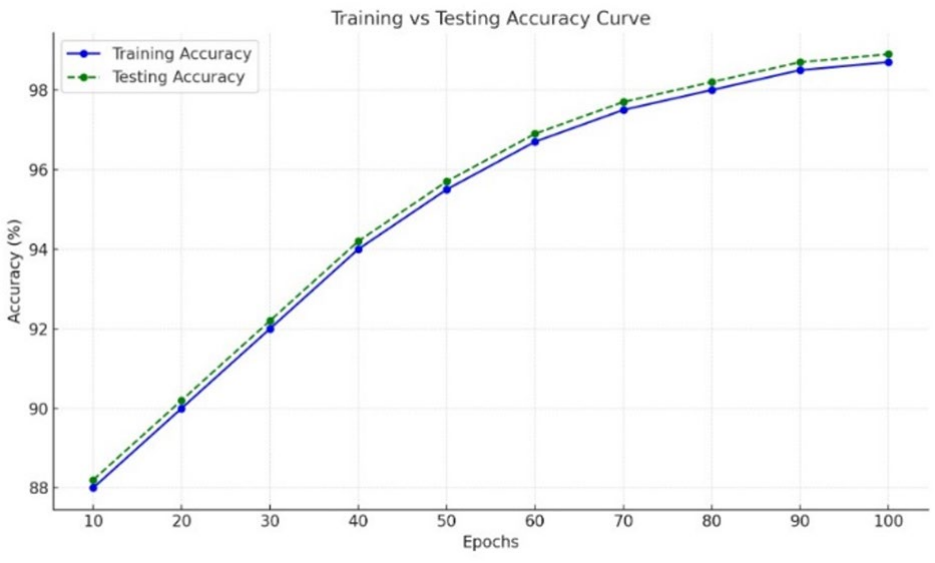
Accuracy Vs epoch of proposed model.

The optimized ensemble learning model have reached accuracy of 0.987 for 100 epochs. Figure 8, represents both training and testing accuracy increase gradually from the start of training, indicates effective model learning. The curves exhibit a steady improvement, which denotes that the model is progressively capturing the underlying patterns in the data. The testing accuracy curve slightly surpasses the training accuracy curve, which is an uncommon but possible occurrence due to regularization, early stopping, or other factors minimizing overfitting. The closeness of the two curves implies that the model generalizes well to unseen data. The initial accuracy starts around 88.1 for training and 0.90 for testing, improving to nearly 0.98 for both by the 100 epochs. The smooth curvature indicates that learning is consistent without significant fluctuations, suggesting a well-tuned model and training process.

## Results and discussion

The performance of the proposed optimized ensemble learning model is evaluated through metrics are accuracy, precision, detection rate and false negative rate given in Eqs. (6)–(9)

6 $$Accuracy=\frac{{TP+TN}}{{\left( {TP+TN+FP+FN} \right)}}$$

7 $$Precision=\frac{{TP}}{{\left( {TP+FP} \right)}}$$

8 $$Detection~rate=\frac{{TP}}{{\left( {TP+FN} \right)}}$$

9 $$FNR=\frac{{FN}}{{\left( {FN+TP} \right)}}$$

To ensure the effectiveness of the proposed method and all the comparative models are evaluated using the stratified k-fold cross validation was employed. Precisely, 10-fold cross validation is performed where the dataset is divided into 10 folds and the originals classes are maintained in each folds. This guarantees that the samples are given proportionally to both the training and testing sets. In each reiteration, the dataset was rationalized and re-partitioned formerly cross-validation, causing in multiple independent valuation runs. The concluding test results correspond to the mean and standard deviation of these metrics across all folds and repetitions. This evaluation strategy suggestively moderates modification caused by random sampling effects and provides a more robust estimate of the model’s generalization proficiency, predominantly for datasets of modest size. This comprehensive validation procedure strengthens the reliability of the performance metrics and supports the practical applicability of the proposed fault detection framework.

### Impact of accuracy on IoT enabled machinery dataset

Accuracy represents the proportion of correct predictions (both true positives and true negatives) relative to the total predictions, playing a critical role in how effectively a system interprets sensor data. Considering the four key factors—temperature, vibration, voltage, and speed—their impact on accuracy is analysed as follows:

(i) Temperature: Variations in temperature leads to sensor drift or inaccurate readings. Extreme temperatures may alter sensor sensitivity or calibration, thereby reducing system accuracy. Ensuring a stable thermal environment helps maintain sensor integrity and improves accuracy by minimizing temperature-induced errors.
(ii) Vibration: High levels of vibration will distort sensor outputs, particularly for precision instruments. Excessive vibrations introduce noise, which compromises the reliability of the data and decreases overall accuracy. Implementing vibration-dampening measures can mitigate this effect and enhance sensor performance.
(iii) Voltage: Fluctuations in voltage disrupts sensor performance, leading to errors in signal transmission or data acquisition. These disruptions undermine system accuracy. Maintaining a stable and consistent voltage supply enhances sensor reliability, directly contributing to improved accuracy.
(iv) Speed: In systems that measure rotational or linear speed, inaccuracies often arise due to miscalibration or noise in the measurements. These errors propagate through the model, negatively impacting overall system accuracy. Proper calibration and filtering techniques are essential to ensure precise speed measurements and maintain accuracy. By addressing these factors, system accuracy is significantly improved with the value 0.984 and ensures reliable interpretation of sensor data and overall system performance.

### Impact of detection rate on IoT enabled machinery dataset

The detection rate, also known as sensitivity, represents the proportion of actual positives accurately identified. It plays a vital role in fault prediction. The influence of detection rate is analysed across the following key factors:

(i) Temperature: Variations in temperature, such as overheating in motors is an early indicator of potential faults. Sensors must be sufficiently sensitive to detect even minor deviations, thereby improving the detection rate and enabling timely interventions.
(ii) Vibration: Abnormal vibrations are often precursors to mechanical failures. Achieving a high detection rate is critical to promptly identify these irregularities. Insufficient sensitivity may result in missed critical warning signs, compromising system reliability.
(iii) Voltage: Voltage fluctuations, including drops or spikes, often indicate power issues that could lead to equipment damage or failure. A high detection rate ensures that such abnormalities are quickly identified, allowing corrective actions to prevent system disruptions.
(iv) Speed: Sudden changes in rotational or linear speed often signal malfunctions in the system. A high detection rate of 0.987 enables the accurate identification of faults and ensures timely responses to prevent further damage or operational inefficiencies.

### Impact of precision on IoT enabled machinery dataset

Precision measures the proportion of correctly identified positives out of all predicted positives, playing a critical role in minimizing false alarms and ensuring system trustworthiness. Its importance is examined across key factors as follows:

(i) Temperature: Minor, non-critical temperature variations can trigger false alarms, disrupting operations. Sensors with high precision effectively differentiate between normal fluctuations and genuine faults, reducing unnecessary interventions.
(ii) Vibration: Precision is crucial in detecting critical vibration thresholds while ignoring benign variations. Excessive false alarms caused by low precision leads to unnecessary shutdowns or corrective actions, reducing system efficiency.
(iii) Voltage: Voltage sensors need to accurately identify problematic fluctuations without being influenced by transient variations also high precision ensures smooth operations by preventing false positives that could unnecessarily disrupt processes.
(iv) Speed: In systems measuring speed, high precision ensures that only significant deviations are flagged. This reduces the risk of false alarms, minimizing unnecessary corrective actions and maintaining system stability. Precision value of 0.981 in detecting faults avoids disruptions caused by false positives and improves the overall efficiency.

### Impact of false negative rate on IoT enabled machinery dataset

False negative rate (FNR) measures the proportion of actual positives that go undetected by the system. Maintaining a low FNR is essential for ensuring safety and reliability. Its impact across key parameters is as follows:

(i) Temperature: Missing critical temperature changes, such as overheating, can result in severe failures or damage. A low FNR ensures the system responds promptly to such changes, preventing catastrophic outcomes.
(ii) Vibration: Undetected abnormal vibrations can significantly increase the likelihood of mechanical failures. A low FNR ensures these issues are identified early, allowing timely interventions to mitigate risks.
(iii) Voltage: Failing to detect voltage anomalies, such as spikes or drops, damage connected devices or disrupt operations. Minimizing the FNR safeguards the system against power-related issues and ensures smooth functionality.
(iv) Speed: Undetected anomalies in speed measurements can cause inefficiencies or accelerated system wear. Reduced FNR rate of 0.02 enhances the system’s ability to identify such faults.

### Comparison of proposed model with existing ML models

The proposed optimized ensemble learning model is compared with other machine learning model such as Decision Tree^41^, Random Forest^42^, Navie Bayes^43^, K- Nearest Neighbour and Decision Stump^44^ depending on the performance metrics are accuracy, precision, detection rate and false negative rate. The proposed model has identified the fault in the IoT enabled machinery and classified those faults as multiclass (class 0 to class 4) shown in Table 5.

**Table 5:** Multiclass w.r.t dataset and its description.

Multiclass	Descriptions
Class 0	Fluctuation in voltage
Class 1	High or low Vibrations
Class 2	Increase or decreases in speed range
Class 3	Change temperature level

**Table 6:** Comparison of proposed model with existing models based on accuracy.

Accuracy				
Learning models	Class 0	Class 1	Class 2	Class 3
Decision Tree	0.932	0.937	0.921	0.927
Random Forest	0.921	0.929	0.924	0.927
Navie Bayes	0.911	0.915	0.920	0.919
KNN	0.925	0.921	0.926	0.930
Decision Stump	0.945	0.947	0.951	0.954
Ensemble model	0.984	0.982	0.986	0.988

In Table 6, the accuracy of the proposed optimized ensemble learning model is high for all class 0 to 4 compared to other models. The proposed model has high accuracy for class 0 (0.984), class 1 (0.982), class 2(0.986) and class 3 (0.988) compared to all other learning models. The decision tree has low accuracy (0.932 to 0.927) due to the high variance that leads to the overfitting. Due to the complexity of the random forest model, it occurs less accuracy (0.921 to 0.927). In case of Navie Bayes, the accuracy (0.911 to 0.919) because of the independent features which are not highly correlated. The accuracy of KNN covers from (0.925 to 0.930), however the choosing the neighbour nodes “k” whether its high or low leads to risk in fault identification. The last model of Decision stump has slight high accuracy (0.945 to 0.954) compared to all models, but low to the proposed model because, it concentrates only on single feature.

**Table 7:** Comparison of proposed model with existing models based on Precision.

Precision				
Learning models	Class 0	Class 1	Class 2	Class 3
Decision Tree	0.926	0.921	0.927	0.920
Random Forest	0.909	0.901	0.908	0.911
Navie Bayes	0.932	0.937	0.939	0.940
KNN	0.942	0.945	0.947	0.949
Decision Stump	0.956	0.962	0.963	0.968
Ensemble model	0.981	0.983	0.985	0.987

In Table 7, the precision of the proposed optimized ensemble learning model has obtained sequential improvement from class 0 to class 1. Class 3 (0.987) has higher precision compared to class 0 (0.981), class 1(0.983) and class 2(0.985) obtained from the proposed ensemble model. The decision tree has lower precision from (0.926 to 0.920) because of complex decision boundaries. In random forest, the less precision occurs from (0.909 to 0.911) due to limited feature. In this data, the features are dominant each other that makes Navie bayes to have a less precision value from (0.932 to 0.940). Usually, KNN is less sensitive to identify the outliers that has less precision value from (0.942 to 0.968). The decision stump also has improved precision value from (0.956 to 0.968) but lesser than proposed model. Thus, the precision of the proposed model ensures the detection of faults in IoT enabled machinery.

**Table 8:** Comparison of proposed model with existing models based on detection Rate.

Detection rate				
Learning models	Class 0	Class 1	Class 2	Class 3
Decision Tree	0.943	0.932	0.938	0.939
Random Forest	0.927	0.922	0.932	0.929
Navie Bayes	0.943	0.947	0.949	0.945
KNN	0.953	0.955	0.957	0.959
Decision Stump	0.961	0.965	0.967	0.969
Ensemble model	0.987	0.988	0.989	0.988

In general, detection rate proves the proposed model for the fault detection in IoT enabled machinery from class 0 (0.987), class 1(0.988), class 2(0.989) and class 3(0.988). Similar to accuracy and precision, the detection rate of the proposed model has higher value compared to decision tree, random forest, navie bayes, KNN, decision stump is shown in Table 8. The false negative rate of the proposed model for classes (0.02, 0.03) defines assumptions of actual faults identification in IoT enabled machinery. Compared to all learning model, the proposed model has less FNR value shown in Table 9.

**Table 9:** Comparison of proposed model with existing models based on false negative rate (FNR).

False negative rate				
Learning models	Class 0	Class 1	Class 2	Class 3
Decision Tree	0.07	0.09	0.07	0.06
Random Forest	0.08	0.06	0.06	0.07
Naive Bayes	0.06	0.08	0.08	0.08
KNN	0.05	0.04	0.05	0.04
Decision Stump	0.04	0.05	0.07	0.06
Ensemble model	0.02	0.03	0.03	0.02

**Table 10:** Comparative results of assessed machine learning models.

Models	Accuracy	Precision	Detection rate	FNR
KNN	0.934	0.928	0.941	0.059
MLP	0.951	0.946	0.954	0.046
AdaBoost	0.962	0.958	0.965	0.035
XGBoost	0.971	0.968	0.973	0.027
ANN	0.948	0.942	0.952	0.048
DNN	0.968	0.964	0.971	0.029
Ensemble Model	0.985	0.984	0.988	0.02

The comparative results in the Table 10. Shows the distinct performance hierarchy for the assessed machine learning models. KNN and Shallow ANN model achieved higher accuracy however the false negative rate for both relies high (0.059 for KNN and 0.018 for ANN) when compared with other models, this shows that the model is not sufficient to detect the subtle fault patterns. MLP and AdaBoost models presents improved performance in terms of accuracy, precision, detection rate and FNR due to its non-linear relationship ability and ensemble decision making process. XGBoost and DNN effectively captures the complex feature interactions, despite all these the proposed models outperform the rest of the models in all the metrics with the score of 0.985 (accuracy), 0.984 (Precision), 0.988 (Detection Rate) and 0.02 (FNR). The significant result of FNR for the proposed model exhibits that it is crucial for industrial fault detection, as missed fault even leads to machine damage, machinery breakdown and fault analysis. Hence the proposed model ensures that the lowest FNR leads to increased productivity with less false rate.

### Discussion on statistical significance and confidence interval analysis

To ensure that the performance of the proposed model is not quantifiable to certain data and to prove its robustness, the confidence interval analysis was carried out with the mean ± standard deviation in terms of accuracy and detection rate. Table 11 presents the detailed comparative analysis of confidence interval analysis with the score of 95%. Mean and standard deviations are computed over various repeated 10-fold cross validation process. From which it is observed that the proposed models exhibit the lowest variance and slenderest confidence intervals to disclose the superior robustness and generalization levels.

**Table 11:** Comparative results of assessed machine learning models.

Models	Accuracy	Accuracy (Mean ± SD)	Detection rate	95% CI	Detection rate (Mean ± SD)	95% CI	Statistical significance
KNN	3.2* 10 − 6	0.934 ± 0.012	2.7 * 10 − 6	[0.910, 0.958]	0.941 ± 0.011	[0.919, 0.963]	Significant
MLP	1.2 * 10 − 5	0.951 ± 0.010	8.6 * 10 − 6	[0.931, 0.971]	0.954 ± 0.009	[0.936, 0.972]	Significant
AdaBoost	4.3 * 10 − 5	0.962 ± 0.009	3.9 * 10 − 5	[0.944, 0.980]	0.965 ± 0.008	[0.949, 0.981]	Significant
XGBoost	6.8 * 10 − 4	0.971 ± 0.008	5.1 * 10 − 4	[0.955, 0.987]	0.973 ± 0.007	[0.959, 0.987]	Significant
ANN	2.4 * 10 − 3	0.948 ± 0.011	1.9 * 10 − 5	[0.926, 0.970]	0.952 ± 0.010	[0.932, 0.972]	Significant
DNN	9.7 * 10 − 4	0.968 ± 0.008	7.8 * 10 − 4	[0.952, 0.984]	0.971 ± 0.007	[0.957, 0.985]	Significant
Ensemble Model	9.8 * 10 − 4	0.985 ± 0.004	7.4 * 10 − 4	[0.977,0.993]	0.988 ± 0.003	[0.982, 0.994]	Significant

The paired t-test was evaluated at a confidence level of 95% with the significance value $\:\:\alpha\:=0.05,\:$using the 10-fold cross validation. From the Table 11 it is evident that all the models are below the significance value and it is computationally significant in terms of accuracy and detection rate which holds the primary role in analysing the machinery faults. The findings confirm that the improvements in performance are not attributed to random fluctuations but are a result of the proposed model’s optimized RPCA-based feature extraction, sequential feature selection, and Bayesian-tuned ensemble learning approach.

In Industrial environments powered by IoT, where edge devices must function within stringent limits on processing power, memory, and energy, computational efficiency is as vital as diagnostic precision for fault detection systems. The RPCA-based optimized ensemble framework introduced here is crafted to emphasize lightweight computation and facilitate real-time application. This method diverges from a standard lightweight deep learning baseline by significantly lowering training complexity through the use of matrix decomposition and shallow learning models, thus bypassing the need for iterative gradient-based optimization across numerous network layers. The model also achieves reduced inference latency by forgoing deep hierarchical feature transformations, instead utilizing low-overhead distance calculations and ensemble voting. Furthermore, the memory requirements of the proposed framework are kept minimal, as it only retains the RPCA components, selected feature subsets, and classifier parameters, unlike deep learning models that necessitate large weight matrices and intermediate activations. These features render the proposed method exceptionally apt for ongoing, real-time fault monitoring in industrial settings.

## Conclusion

The experimental results of the proposed optimized ensemble learning model demonstrate the model’s efficacy in fault detection, achieving remarkable performance metrics with an accuracy of 0.984, precision of 0.981, a detection rate of 0.987, and a low false negative rate of 0.02. These metrics underscore the robustness and reliability of the proposed framework, particularly in identifying faults accurately by ensuring the high detection with minimal false negative rate. By addressing the challenges of noisy data and high-dimensional sensor inputs, the optimized robust PCA method proves the quality of feature extraction, while the ensemble model provided the adaptability and predictive strength required for real-world fault diagnosis in industrial systems. Despite its promising results, the model also opens several avenues for future exploration to advance fault detection in IoT-enabled infrastructures. Explainability is another crucial aspect that warrants attention. Integrating explainable AI techniques would make the fault detection process more transparent, enabling operators to understand the reasoning behind the model’s predictions. This not only fosters trust but also aids in diagnosing root causes more effectively. Predictive maintenance capabilities could also be integrated into the system to transition from reactive to proactive fault management, leveraging time-series data to anticipate and mitigate faults before it occurs. The assessment primarily depends on simulated data, which may not fully reflect real-world complexities such as sensor degradation and communication problems, thus affecting its generalizability. The fault injections adhere to predefined patterns that may not encompass the entire spectrum of industrial fault behaviours. Future research will involve validating the model with actual industrial data, investigating online learning, and evaluating constraints related to practical deployment.

## Data Availability

The datasets generated during and /or analysed during the current study are available from the corresponding author upon reasonable request.
